# Differences in Remote Mental Healthcare: Minority Ethnic Service User Experiences and Perceptions During COVID-19

**DOI:** 10.1192/bjo.2022.162

**Published:** 2022-06-20

**Authors:** Lamiya Samad, Bonnie Teague, Karen Moreira, Sophie Bagge, Khalifa Elzubeir, Emma Marriott, Jonathan Wilson, Nita Agarwal

**Affiliations:** 1 Norfolk and Suffolk NHS Foundation Trust, East of England, United Kingdom; 2 UCL Great Ormond Street Institute of Child Health, London, United Kingdom; 3 University of East Anglia, East of England, United Kingdom

## Abstract

**Aims:**

COVID-19 has resurfaced health inequalities but also provides new opportunities for remote healthcare. Minority ethnic service users (SUs) are substantially under-represented in secondary mental health services due to gaps in understanding needs of this priority group. We aimed to assess and identify any differences in characteristics and acceptability, with a focus on minority ethnic mental health SUs.

**Methods:**

A prospective, online feedback questionnaire was developed with the help of SUs. This was built into video consultations (VCs), using the secure Attend Anywhere platform through a survey link. We present results between July 2020 and January 2022, during which, a total of 2,565 SUs completed the online questionnaire after VCs. SPSS (version 27) was used for descriptive statistical analysis. Chi-squared test, using 5% level of significance, was conducted to test differences between the two (minority Vs majority ethnic) SU groups.

**Results:**

Of 2,565 SUs, 119 (4.6%) were from minority ethnic groups (Asian British, Mixed/multiple, Black British, and Other), 2,398 (93.5%) were White British, and 48 (1.9%) preferred not to disclose. A higher percentage of SUs were females from both minority (55.6%) and White British (66.1%) ethnic groups (ϰ^2^=5.476, *p* < 0.05). By age group, almost half (48.7%) of minority ethnic SUs were less than 25 years old, compared with those from White British ethnicity (29.2%). In contrast, only 2.5% minority ethnic SUs were aged ≥65 years with none ≥80 years old (ϰ^2^ Likelihood Ratio = 27.11, *p* < 0.001).

No significant differences were found for video technical quality, such as waiting area, joining the video call, sound, and video quality. Similar findings were observed for video care delivery aspects with no significant differences between (minority ethnic and White British) SUs. Overall, both groups felt comfortable during the video call (ϰ^2^=0.137, *p* > 0.05), their needs were met (ϰ^2^=0.384, *p* > 0.05) and felt supported (ϰ^2^=0.164, *p* > 0.05). However, according to care team, a significantly higher percentage of minority ethnic SUs (43%) had remotely consulted Specialist (Eating disorders, Well-being/IAPT) services compared with those of majority ethnicity (29%) (ϰ^2^ Likelihood Ratio = 21.936, *p* < 0.05).

**Conclusion:**

Both minority ethnic and White British SUs found video care to be acceptable, with positive experiences. A significantly high proportion of minority ethnic SUs was younger and had remotely consulted Specialist services, with none in the 80-plus age group. These findings highlight priority areas to address among this massively underrepresented group in mental healthcare services.

